# Are all sugars equal? Role of the food source in physiological responses to sugars with an emphasis on fruit and fruit juice

**DOI:** 10.1007/s00394-024-03365-3

**Published:** 2024-03-16

**Authors:** Javier T. Gonzalez

**Affiliations:** 1 Centre for Nutrition, Exercise and Metabolism, University of Bath, Bath, UK; 2 Department for Health, University of Bath, Bath, BA2 7AY UK

**Keywords:** Food matrix, Glycaemia, Health, Metabolism, Sugars

## Abstract

High (free) sugar intakes can increase self-reported energy intake and are associated with unfavourable cardiometabolic health. However, sugar source may modulate the effects of sugars due to several mechanisms including the food matrix. The aim of this review was to assess the current state of evidence in relation to food source effects on the physiological responses to dietary sugars in humans relevant to cardiometabolic health. An additional aim was to review potential mechanisms by which food sources may influence such responses. Evidence from meta-analyses of controlled intervention trials was used to establish the balance of evidence relating to the addition of sugars to the diet from sugar-sweetened beverages, fruit juice, honey and whole fruit on cardiometabolic outcomes. Subsequently, studies which have directly compared whole fruit with fruit juices, or variants of fruit juices, were discussed. In summary, the sources of sugars can impact physiological responses, with differences in glycaemic control, blood pressure, inflammation, and acute appetite. Longer-term effects and mechanisms require further work, but initial evidence implicates physical structure, energy density, fibre, potassium and polyphenol content, as explanations for some of the observed responses.

## Introduction

**Fig. 1:**
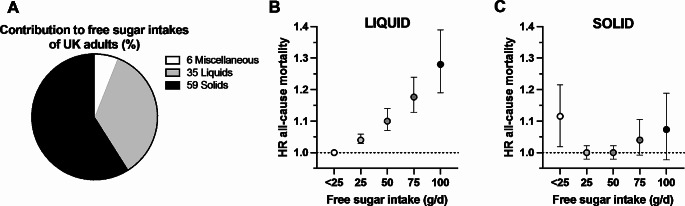
Contribution of different food physical structures to free sugar intakes in UK adults, and the associations between physical structure of free sugars and mortality. Data in panel **A** are from the National Diet and Nutrition Survey Panel [1] and in panels **B** and **C**, from Kaiser et al. [2]

Free sugars are commonly consumed in a variety of foods differing in various physical forms (Fig. 1A) [1]. Yet little is known about how physical form and other aspects of the food matrix impact the cardiometabolic health responses to sugars. Observational data demonstrate that increasing consumption of free sugars from liquids is positively (unfavourably) associated with all-cause mortality (Fig. 1B) [2], whereas there is little evidence that ingesting free sugars from solids is associated with mortality (Fig. 1C) [2]. Therefore, the physical form in which free sugars are ingested in, such as liquid, semi-solid, or solid, may moderate the health effects of sugars [3, 4]. Whilst these observational data provide an important rationale to investigate effects of food form, they cannot alone establish causality and have the potential to mislead [5, 6]. Consequently, there is a need to establish the causal effects of food physical form on the metabolic and health responses to free sugars. Furthermore, within liquid sources of sugars, there is potentially additional moderation of the effects of sugars by other aspects of food matrix, which can lead to greater complexity. This greater complexity can also lead to confusion and inconsistency in recommendations. For example, the recommendations for fruit juices to be included or excluded as part of the recommended diet vary by country [7]. There is, therefore, a need to better understand the role of food matrix on the physiological responses to sugar sources. This will improve understanding of which sources may be likely to produce more favourable or less favourable health effects and provide an opportunity to improve the health profile of sugar-containing foods.

In addition to being a potential area of confusion, products derived from whole fruit, such as puree and juice, are a useful example to understand food matrix effects on the physiological responses to sugars since they contribute to understanding the interactions of different aspects of the food matrix (e.g., physical structure *versus* nutrient composition). Furthermore, fruit juice provides an interesting food for comparisons against other sugar-containing liquids since fruit juice intake does not show the expected positive relationship with mortality seen for other sugar-containing liquids [2], and is in fact negatively associated with stroke risk [7]. Hence, specific factors within fruit juices may modulate the physiological responses to ingestion when compared with other liquid sources of sugars. Orange and apple juices dominate the UK market share of fruit juices [8] and will therefore be used as primary examples within this review. It should also be recognised that most associations or effects of fruit juices versus other liquid sugars are likely to only hold for 100% fruit juice. These will be compared with their whole-fruit counterparts, and to other key sources of sugars in more simple food matrices [i.e., sugar-sweetened beverages (SSBs) and honey]. Other sugar sources within solid foods, such as cakes, biscuits, and confectionary, will be discussed in passing where relevant but will not be a primary focus due to the large heterogeneity in nutrient composition and physical structure of such foods.

It should be noted that understanding the individual causal mechanisms which can explain an observed effect of food matrix on a physiological response can be difficult to disentangle. This is largely due to the potential interacting factors of the components which contribute to the food matrix, producing neutral, additive, synergistic or inhibitory interactions. For example, ingestion of fruit smoothies with a high polyphenol oxidase content such as banana-based smoothies have been demonstrated to decrease the bioavailability of certain dietary polyphenols when compared to ingestion of fruit smoothies with a low polyphenol oxidase content such as berry-based smoothies [9]. Furthermore, understanding the kinetics of digestion, absorption and metabolism of carbohydrates with complex food matrices has technical and financial viability challenges [10].

The aim of this review is to assess the current state of evidence in relation to food source effects on the physiological responses to dietary sugars in humans with a focus on fruit and fruit juice. The physiological responses discussed will include those which play either a direct or indirect role in cardiometabolic health, such as blood glucose and insulin sensitivity, blood lipids and inflammation, gastric emptying and appetite, and blood pressure and vascular function. The potential mechanisms by which food matrix may influence such responses will be discussed which include physical structure, polyphenol, fibre, fat and water content, and sugar composition.

## Methods

For the primary focus of this review, PubMed was searched for meta-analyses of human controlled intervention trials investigating the effects of sugar sources on cardiometabolic outcomes. The sources to be considered were sugar-sweetened beverages, fruit juice, honey, and whole fruit. Where meta-analysed data from controlled trials were available, these were only used when at least 3 studies comprising the meta-analysed data to provide more conservative inferences.

### What are the key nutritional differences between sugars sources?

With the exception of honey, when expressed per 100 g of food or per 100 mL of fluid, there is little difference between sugar sources in the energy or macronutrient content (Table 1). However, there are some notable differences in fibre, potassium, and polyphenol content (Table 1) [3, 11, 12]. In particular, 100% fruit juices and whole fruit display higher fibre, potassium and polyphenol content than SSBs and whole fruit contain more fibre than fruit juices. Whilst honey appears to have a high potassium and polyphenol content when expressed per 100 g, it is arguably more relevant to interpret these per g of sugar. When the nutritional composition is expressed per g of sugar, fruit juices - and especially whole fruit - display a markedly higher fibre, potassium, and polyphenol content than both SSBs and honey (Table 1). The potential relevance of these differences will be discussed after an overview of the evidence regarding the physiological effects of these sugar sources. It should also be noted that the portion size of these sugar sources varies and can therefore alter the likely intakes of nutrients and the glycaemic load (Table 2).

**Table 1:** Nutrient composition of sugar sources expressed per g or per mL of food item and as per g sugar

	SSB	Honey	Orange Juice (no pulp)	Apple Juice (clear)	Orange Juice (with pulp)	Apple Juice (cloudy)	Orange (whole fruit)	Apple (whole fruit)
**Energy** (kcal/100 g or kcal/100 mL)	42	307	38	42	41	46	43	60
**Fat** (g/100 g or g/100 mL)	Negligible	Negligible	Negligible	Negligible	Negligible	Negligible	Negligible	0.5
**Carbohydrate** (g/100 g or g/100 mL)	11	76	7.9	10	9.3	11	8	13
**Sugars** (g/100 g or g/100 mL)	11	76	7.9	9.6	8.6	10	8	11
**Protein** (g/100 g or g/100 mL)	Negligible	0.5	0.6	Negligible	0.8	0	0.8	0.6
**Fibre** (g/100 g or g/100 mL)	Negligible	Negligible	0.1	Negligible	0.3	0.7	1.2	1.2
**Potassium** (mg/100 g or mg/100 mL)	2	130	150	90	150	90	90	90
**Polyphenols** (mgGAE/100 g or mgGAE/100 mL)	Negligible	56–246	∼ 210	∼ 150	∼ 230	∼ 200	∼ 230	∼ 290
**Fibre** (g/g sugar)	Negligible	Negligible	0.01	Negligible	0.03	0.07	0.15	0.11
**Potassium** (mg/g sugar)	Negligible	2	19	9	17	9	11	8
**Polyphenols** (mg/g sugar)	Negligible	1–3	∼ 27	∼ 16	∼ 27	∼ 20	∼ 29	∼ 26
**Glycaemic index**	63 (moderate)	60 (moderate)	48 (low)	33 (low)	48 (low)	37 (low)	45 (low)	44 (low)
**Glycaemic load**								

GAE, gallic acid equivalents. Data from [3, 4, 11–14]. Data are means values reported in cited studies or provided as a single value obtained from a database, ∼ denotes substantial potential for variation

**Table 2:** Nutrient composition of sugar sources expressed per UK portion size

	SSB	Honey	Orange Juice (no pulp)	Apple Juice (clear)	Orange Juice (with pulp)	Apple Juice (cloudy)	Orange (whole fruit)	Apple (whole fruit)
**UK Serving size** **(mL or g)**	330 mL	5 g	150 mL	150 mL	150 mL	150 ml	110 g	110 g
**Energy** (kcal)	139	15	57	63	62	69	47	66
**Fat** (g)	Negligible	Negligible	Negligible	Negligible	Negligible	Negligible	Negligible	0.6
**Carbohydrate** (g)	36	3.8	12	15	14	17	9	14
**Sugars** (g)	36	3.8	12	15	13	15	9	12
**Protein** (g)	Negligible	Negligible	0.9	Negligible	1.2	0	0.9	0.7
**Fibre** (g)	Negligible	Negligible	0.2	Negligible	0.5	1.1	1.3	1.3
**Potassium** (mg)	7	7	225	135	150	100	100	90
**Polyphenols** (mgGAE)	Negligible	3–12	∼ 315	∼ 225	∼ 345	∼ 300	∼ 345	∼ 290

GAE, gallic acid equivalents. Data from [3, 4, 11–14]. Data are means values reported in cited studies or provided as a single value obtained from a database, ∼ denotes substantial potential for variation

### Sugar sources and the relationships to cardiometabolic health

#### Blood glucose and insulin sensitivity

**Fig. 2:**
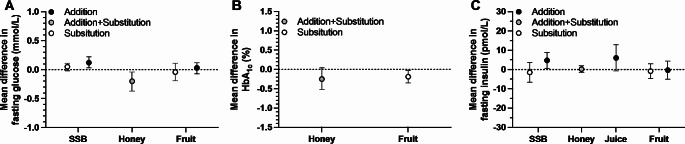
Effects of experimental addition and/or substitution of various sugar sources into the diet on fasting glucose concentrations, glycated haemoglobin (HbA_1c_) and fasting insulin concentrations. Data are mean differences ± 95%CI redrawn from Ahmed et al. for honey [15] and Choo et al. for all other food sources [16]. SSB, sugar-sweetened beverages. Fructose-containing sugar doses in Choo et al. was a median of 15% energy intake for 4.5 weeks in substitution trials, and 12.2% energy intake for 6 weeks in addition trials. Honey doses in Ahmed et al. were at a median of 40 g of honey for 8 weeks

The glycaemic index of SSBs and honey are both in the moderate range, whereas the glycaemic index of orange and apple juice, and whole oranges and apples are all in the low range (Table 1) [13], suggesting that the addition and/or combination of factors in fruit-sources of sugars can lower the immediate glucose response when normalised for the amount of carbohydrate ingested. This acute response, however, does not seem to directly translate into chronic responses, whereby meta-analyses suggest that honey consumption can lower fasting glucose concentrations [15] (Fig. 2A). Whilst less clear than with acute responses, data do still show a broad pattern which is consistent with a role of food matrix effects on glycaemic control, such as an increase in fasting glucose concentration with addition of excess energy from liquid sources of sugars such as SSBs, which is not seen with addition of whole fruit (Fig. 2A) [16]. Indeed, substitution of whole fruit for other sources of energy in the diet can reduce HbA_1c_ by ∼ 0.19% (95%CI: -0.03 to -0.35%; Fig. 2B) [16].

Glycaemic control is largely influenced by insulin secretion and insulin sensitivity. In people with normal β-cell function, for a given glucose concentration, higher insulin concentrations can be a marker of lower insulin sensitivity. Therefore, fasting insulin concentrations are often used marker of insulin sensitivity. Meta-analyses demonstrate that addition of excess energy as SSBs, but not substitution, can increase fasting insulin concentrations by 5 pmol/L (95%CI: 1 to 9 pmol/L; Fig. 2C) [16]. There is some indication that the source of sugars may be important in this regard, since evidence does not indicate that addition of fruit increases fasting insulin concentrations (mean difference − 0.3 pmol/L; 95%CI: -5 to 4 pmol/L) [16].

Direct comparison between sugar sources on glycaemic control and insulin sensitivity has been performed in several studies, albeit as secondary or tertiary outcomes [3, 4]. When intake of two whole apples per day for 8 weeks was compared to a sugar-matched control beverage made from apple juice, the treatment effect on fasting glucose and insulin concentrations was − 0.06 mmol/L (95%CI: -0.15 to 0.03 mmol/L) and − 0.05 pmol/L (-0.10 to 0.00 pmol/L), respectively [3]. Furthermore, when intake of 550 g/d whole apples was compared to 500 mL of either clear or cloudy apple juice, the changes in insulin concentrations were − 3 ± 17 pmol/L with whole apples, 8 ± 10 pmol/L with cloudy apple juice, and 3 ± 11 pmol/L with clear apple juice (treatment effect *p* > 0.05) [4]. Taken together these data indicate that the source of sugars may play a role in glycaemic control, whereby fruit sources, and in particular whole fruit may improve glycaemic control and insulin sensitivity, when compared with mixed comparators. These data are consistent with observational evidence demonstrating negative associations of whole fruit intake with development of diabetes, and a neutral association with 100% fruit juice [17–19]. Evidence from direct comparisons between sugar sources, however, is limited and therefore the certainty of causality between the food matrix effects of sugars on glycaemic control is constrained.

#### Blood lipids, lipoproteins, and inflammation

Low-density lipoprotein cholesterol (LDL-c) and chronic systemic inflammation are central drivers of atherosclerotic cardiovascular disease (CVD) [20]. Meta-analyses indicate that high fructose intakes can increase plasma apolipoprotein B and triglyceride concentrations during hypercaloric feeding trials, but evidence does not indicate such increases during isocaloric feeding trials [21]. When sources of sugars have been directly compared, consuming two whole apples per day for 8 weeks has been shown to reduce LDL-c concentrations by 0.14 mmol/L (0.02 to 0.26 mmol/L) and fasting triglyceride concentrations by 0.05 mmol/L (0.01 to 0.08 mmol/L) when compared with a sugar-matched control beverage comprised of fruit juice [3]. Somewhat consistent with this, consumption of 550 g apples per day for 4 weeks lowered LDL-c concentrations by > 0.3 mmol/L compared with 500 mL of clear apple juice per day, with similar LDL-c reductions when whole apples were compared with cloudy apple juice. The evidence did not indicate any significant differences in triglyceride response between intake of whole apples (-0.06 ± 0.38 mmol/L) compared with clear (0.03 ± 0.34 mmol/L) or cloudy (0.01 ± 0.36 mmol/L) apple juice [4].

**Fig. 3:**
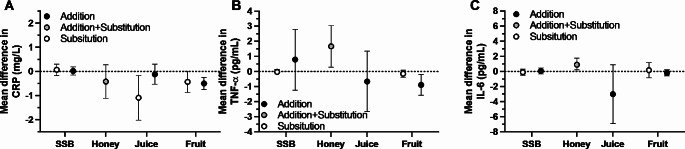
Effects of experimental addition and/or substitution of various sugar sources into the diet on circulating inflammatory marker concentrations. Data are mean differences ± 95%CI redrawn from Ahmed et al. for honey [15] and Qi et al. for other food sources [22]. SSB, sugar-sweetened beverages. Fructose-containing sugar doses in Choo et al. was a median of 9% energy intake for 6 weeks in substitution trials, and 8% energy intake for 5 weeks in addition trials. Honey doses in Ahmed et al. were at a median of 40 g of honey for 8 weeks

The role of sugar source may also play a role in inflammatory marker responses to sugar intake. Meta-analysis of sugar sources demonstrates that C-reactive protein (CRP) concentrations are not lowered by either substitution or addition of SSBs [22]. However, whole fruit can lower CRP with either substitution or addition to the diet [22]. Similarly, whereas the data did not support a decrease in TNF-α concentrations with addition of SSBs or fruit juice to the diet, addition of whole fruit can lower TNF-α concentrations (Fig. 3B) [22]. Finally, for interleukin-6 (IL-6), the evidence did not suggest that SSBs, fruit juice, or whole fruit increased or decreased IL-6 concentrations (Fig. 3C) [22]. Interestingly, honey intake was demonstrated to increase IL-6 concentrations [15]. Direct comparisons of sugar sources do not provide evidence that either CRP or TNF-α concentrations differ with addition of whole fruit compared to fruit juice [3, 4]. Since energy balance status can influence inflammatory markers and possible mask potential effects of a dietary intervention, it is notable, that substitution studies with fruit juice were performed in either neutral or negative energy balance [22], whereas both substitution and addition studies of whole fruit were performed in either neutral or positive energy balance [22]. This suggests that these effects of fruit juice and of whole fruit can be seen within the context of changes in energy balance.

Accordingly, there is good evidence that the source of sugars can influence circulating LDL-c responses, whereby a more complex, whole/intact, food source can lower LDL-c concentrations compared with simpler, processed sources of sugars. Effects on triglyceride concentrations are less consistent, as are effects on inflammatory markers, with some suggestions of potential for fruit juice or whole fruit to lower some circulating inflammatory markers, albeit with less direct evidence.

#### Blood pressure and vascular function

**Fig. 4:**
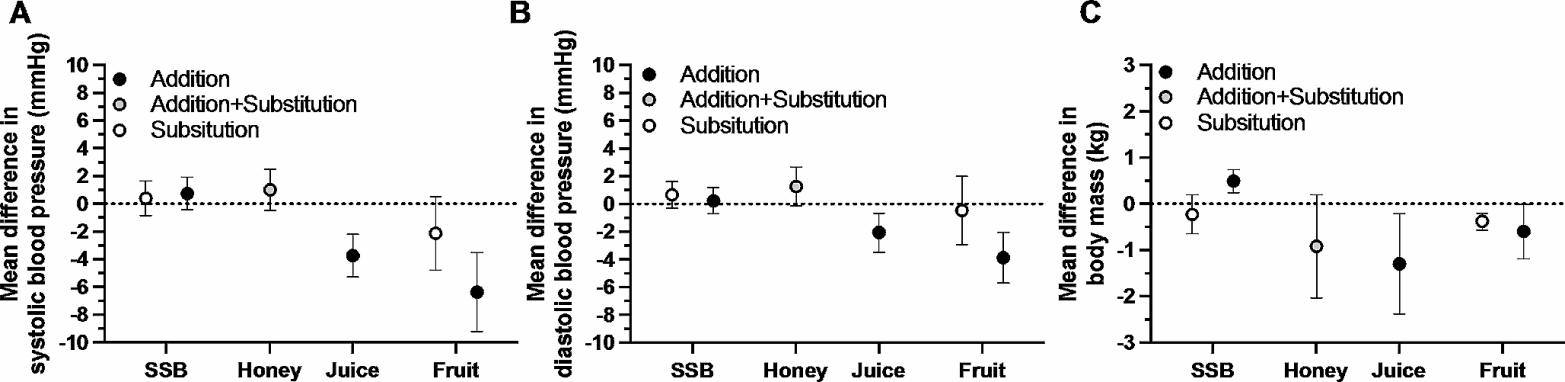
Effects of experimental addition and/or substitution of various sugar sources into the diet on systolic blood pressure, diastolic blood pressure and body mass. Data redrawn from Ahmed et al. for all outcomes with honey [15], Qi et al. for blood pressure outcomes with other food sources [25], and Chiavaroli et al. for body mass with other food sources [26]

Blood pressure and vascular function play a major role in cardiometabolic health [23, 24]. Meta-analyses of sugar sources demonstrates that substitution or addition of SSBs or honey to the diet do not lower either systolic or diastolic blood pressure, whereas the addition of either fruit juice or whole fruit can lower both systolic and diastolic blood pressure (Fig. 4A and B) [15, 25]. Direct comparison of fruit sources does not provide evidence of differences between whole fruit compared with fruit juice consumption on either systolic or diastolic blood pressure [4]. Accordingly, it may be possible to achieve the blood pressure lowering effects of fruit from either fruit juice or from whole fruit.

Vascular structure and function play a key role in cardiometabolic health and blood pressure regulation. Changes in each of three layers of the artery can regulate vascular structure and/or function. These include the central and peripheral arterial stiffness of the tunica adventitia, captured by pulse wave velocity, distensibility and β-stiffness [27], smooth muscle function of the tunica media, captured by nitroglycerine-mediated dilation [28], and endothelial function of the tunica intima, captured by flow-mediated dilatation [29]. Changes in vascular function can influence cardiometabolic health in several ways. These include glycaemic control via delivery of insulin and glucose to skeletal muscle, and regulation of blood pressure via the relationship between blood flow, vascular resistance, and blood pressure.

Direct comparison of sugar sources has demonstrated some effects on markers of vascular function in healthy people. For example, consumption of 200 mL per day of orange juice for 2 weeks increased flow-mediated dilation compared with a SSB, without a detectable change in blood pressure [30]. Furthermore, consumption of 500 mL orange juice per day for 4 weeks lowered diastolic blood pressure by ∼ 5 mmHg compared with equivalent ingestion of a SSB, and that the addition of the flavonoid hesperidin (∼ 300 mg, equivalent to 500 mL orange juice) to the SSB can also lower diastolic blood pressure by ∼ 5 mmHg relative to placebo [31]. Whilst no evidence of chronic changes in vascular function were observed when measured in the overnight fasted state, acute increases in postprandial microvascular endothelial reactivity were observed with both orange juice and a hesperidin-fortified beverage versus a SSB [31]. The endothelium-dependent microvascular vasodilatory response to acetylcholine has also been shown to increase with supplementation of two whole apples per day for 8 weeks, compared with an apple juice-based control beverage, alongside a reduction in intracellular cell adhesion molecule-1 (ICAM-1), although no evidence for differences in other adhesion molecules was observed [3].

There is consistent evidence that the source of sugars can influence blood pressure and vascular function with some effects apparent within hours of consumption. Pure fruit juices, particularly orange, grapefruit and grape juice, and whole fruit show generally favourable responses, such as lower blood pressure and increases in flow mediated dilatation and microvascular endothelial reactivity which are not observed with SSBs and honey. Further improvements in endothelial function have been observed with whole fruit, yet this did not yield further reductions in blood pressure.

#### Appetite and energy intake

Long-term changes in body weight and fat mass primarily reflect energy balance, that is, energy intake minus energy expenditure. Therefore, effects of sugar sources on appetite and energy intake have implications for the regulation of body mass. The control of appetite and energy intake is complex and comprises many factors. However, a primary driver of energy intake is energy density [32]. Diets high in free sugars have been shown to increase self-reported energy intake [33], which is likely to be largely explained by the energy density of the diet. Recently, additional factors have been suggested to play a role such as the degree of food processing. Interestingly, according to a commonly used version of the NOVA (not an acronym) classification system, “carbonated drinks” are classified as group 4 (“ultra-processed”), whereas fruit juices are classified as group 1 (“unprocessed or minimally processed”) despite having very similar energy and sugar contents (Tables 1 and 2) [34]. Consequently, US portions of fruit juice, equivalent to 240 mL, end up providing more energy and sugars than does a portion of fruit, yet the difference is smaller with UK portion sizes (Table 2). When this classification is considered in light of some evidence that largely ultra-processed diets can increase energy intake and body mass compared with largely unprocessed diets matched for presented energy, energy density, macronutrients, sugar, sodium and fibre [35], this may have relevance for sugar sources, appetite and energy intake. Nevertheless, is should be noted that in the only current RCT of ultra-processed diets on body mass, energy density of foods was higher in the ultra-processed condition and therefore, there is a need to understand whether ultra-processed foods increase energy intake independent from energy density. Furthermore other evidence suggests that factors such as food texture and physical structure may be at least as important as processing [36, 37], and these factors - alongside energy density - are a more objective, and thus operationally useful way of characterising foods than the NOVA system [37].

When evidence from RCTs of addition of sugar calories or substitution of sugar calories in the diet are examined, the source of sugar may modulate effects on body mass. Indeed, meta-analyses indicate that addition of SSBs can increase body mass, whereas addition of either fruit juice or whole fruit can lower body mass (at least when comprising < 10% of total energy intake; Fig. 4C) [26]. It should, however, be noted that the wide confidence interval for the effect of fruit juice on body mass means this effect size should be interpreted somewhat cautiously until more evidence is available. Notwithstanding this, in direct comparisons of whole fruit with fruit juice, there is no evidence for a difference in the body mass responses with whole apples compared with apple juice a juice-based control beverage [3, 4].

One of the first studies to directly compare sugar sources on appetite found that ingestion of whole apples resulted in a higher satiety rating compared with apple puree and apple juice, although the effects were short-term and no longer apparent after 2 h [38]. Direct comparisons of fruit sources of sugars with SSBs are rare. However, when examined in isolation, SSBs preloads do not normally produce any compensation in subsequent energy intake, thereby tending to produce passive overconsumption [39]. Recent, direct comparisons of apples in different forms, such as whole fruit *versus* puree *versus* juice, consistently find that whole apples result in greater satiety ratings than apple juice [40, 41]. Furthermore, when these were tested in a preload-test meal design to assess energy intake, apple juice (with or without fibre) preloads resulted in compensation such that total energy intake (preload plus test meal) did not differ from control. In contrast, apple sauce lowered total energy intake, which was reduced further still by whole apples [40]. Consistent with this consumption of a mixture of fruits consumed in liquid (apple and grape juice) *versus* solid (apple, grapes, and raisins) form, results in weaker acute satiation and satiety responses, particularly in people with overweight/obesity, although differences in appetite ratings were not detected after 8 weeks of supplementation. Somewhat consistent with this, the addition of ∼ 500 kcal of fruit and vegetables to the diet for 8 weeks resulted in increases in body mass in the region of 1.5-2 kg, with the difference between consumption as liquids versus solids being 0.6 ± 14.9 kg (mean ± SD, *p* = 0.19) [42]. These data suggest that extrapolation from acute appetite responses to longer-term changes in body mass requires caution [43]. Taken together, these data suggest that sugar source can influence appetite and energy intake responses in the short term, while meta-analyses suggest that sugar source can make a difference to body mass. However, direct translation from acute appetite responses to longer-term changes in body mass is not warranted.

### Potential mechanisms by which sugar sources influence cardiometabolic health

The potential mechanisms by which sugar source may influence the physiological responses described above could include oral processing, gastric emptying, digestion and intestinal absorption rates, sodium/potassium balance, modulation of the gut microbiome, and/or alterations in appetite-related gut hormones. The properties of sugar sources which could modulate these mechanisms include the physical structure, and the content and type of carbohydrates, fibre, polyphenols, fats, proteins, water and micronutrients within the food or beverage. These properties will be discussed in relation to the potential mechanisms which may mediate the physiological responses to sugar sources.

#### Oral processing, gastric emptying and intestinal absorption

The physical structure of a food (liquid versus solid, and textures of solid and semi-solid foods) affect bite size, number of chews per bite, and the duration of oro-sensory exposure [44]. In turn these responses can affect rates of eating and energy intake. Faster eating rates are associated with increases in total energy intake within a meal, which can contribute to the ways in which the physical structure of a food can influence overall energy intake. Solids are typically consumed more slowly than semi-solids and liquids (10–120 g/min versus up to 600 g/min) [45, 46], and within solid foods, harder foods are typically consumed more slowly than softer foods [47].

Gastric emptying rates could play a key role many of the effects of sugar source on health outcomes including reductions in blood glucose and increases in satiety. Slower gastric emptying can slow down the rate of nutrient delivery to the intestine and thereby contribute to slower intestinal absorption rates. In turn, this can be one mechanism by which postprandial glucose and/or insulin concentrations are lowered. However, one consideration is that, despite differences in rates of digestion and absorption, some foods can still elicit similar postprandial glucose concentrations, as increases in glucose appearance rates can be offset by increases in insulin-stimulated glucose clearance rates [48]. This may, in part, explain why the glycaemic index of fruit juices and whole fruit are reported as broadly similar despite the former being classed as a source of free sugars (Table 1). Indeed, when whole apples and apple juice were directly compared, apple juice produced a higher postprandial insulin response, in the presence of a similar peak glucose concentration [38]. Using magnetic resonance imaging (MRI) it has been shown that gastric emptying rates are slower with whole apples (half-life: ∼65 min) when compared with either apple puree (∼ 41 min) or apple juice (∼ 38 min) [41]. Unfortunately, plasma glucose kinetics in response to whole fruit *versus* fruit juice have - to date - never been assessed, which may be due to technical challenges with tracer labelling of whole foods [10].

Slower intestinal absorption rates could affect health in more ways than just the acute glucose and insulin response. When fructose-containing sugars are ingested, the fructose can be converted by the intestine and liver into glucose, glycogen, lactate and triglycerides [49, 50]. The partitioning between these metabolic fates may depend on the rate of fructose delivery to the intestine and liver. In mice, for example, a slower delivery of fructose (4 × 0.5 g/kg *versus* a single bolus of 2 g/kg) lowers *de novo* lipogenesis by almost 50% [51]. This lowering of *de novo* lipogenesis with slow fructose delivery might relate to the fact that rodent intestine can convert much of the fructose into other metabolites, but this interconversion can be saturated, leading to more spillover of fructose to the liver when ingested rapidly [51]. *De novo* lipogenesis is an important process in the regulation of hepatic lipid content and atherogenic lipoprotein production [52]. Therefore, if these responses are conserved in humans [53], a slower delivery of fructose to the intestine and liver may contribute to lowering blood lipids.

A slower gastric emptying and intestinal absorption rate from difference sugar sources could be explained by the combined effects of physical structure [41], and nutrient composition (Table 1). Dietary fibre and polyphenol content are two of the most likely components to influence gastric emptying and/or intestinal absorption rates of sugars. Soluble fibres such as pectin can increase the viscosity of food/fluids. The increase in viscosity is thought to be a primary mechanism by which gastric emptying rates and intestinal absorption rates are slowed, since disruption of viscosity by hydrolysis abolishes the effects of soluble fibres on slowing gastric emptying rates and reducing postprandial glycaemic excursions [54]. Nevertheless, removal of fibre naturally present in solid foods can still increase gastric emptying rates [55], demonstrating that the role of fibre content in gastric emptying is relevant across the range of food physical structure.

In addition to delaying gastric emptying, viscous dietary fibres could also slow sugar absorption rates via inhibition if digestive enzyme activity, reduced diffusion of end products of digestion to the intestinal microvilli and/or the generation of a barrier to absorption at the mucosa [56]. It is unclear, however, whether the dose of fibres within fruit is, alone, enough to explain the acute glucose lowering effects of fruit. For example, a meta-analysis reported that 10 g pectin may be required to elicit a reduction in peak postprandial glucose concentrations [57], yet a large apple typically contains less than 5 g pectin [4]. A slower glucose *flux*, however, may be present without an observable change in glucose *concentration* [48], and hence the dose of pectin required to slow sugar absorption rates (and thus sugar flux) may be lower than 10 g, although this remains to be established. Whilst the dose of pectin required to lower postprandial glucose concentrations seems unlikely to be consumed in a single portion of normal fruit, evidence does indicate that the satiety effects of pectin could occur at lower doses, since as little as 5 g of pectin has been shown to increase satiety when added to orange juice [58]. Notably, not only may soluble viscous fibre play a role in the satiety responses of sugar ingestion but may also contribute to the blood pressure lowering effects seen for whole fruit. Meta-analysis indicated that a median intake of 8.7 g soluble viscous fibre per day for 7 weeks can lower systolic blood pressure by 1.6 mmHg (95%CI: 0.5 to 2.7 mmHg) and diastolic blood pressure by 0.4 mmHg (95%CI: 0.01 to 0.8 mmHg) [59].

As well as fibre content, polyphenol content could also contribute to slowing sugar absorption rates, possibly via reducing digestive enzyme activity (e.g., sucrase) [60] and/or inhibiting intestinal sugar transporters (e.g., SGLT1 and GLUT5) [61, 62]. Consistent with this, apple polyphenol-rich drinks can lower postprandial glucose concentrations in humans without altering gastric emptying rates as assessed by the paracetamol test [63]. The glucose lowering in response to apple polyphenols demonstrated a dose response (essentially linear) up to at least 935 mg catechin equivalents (per 200 mL dose). With a reduction seen with as little as ∼ 450 mg catechin equivalents (per 200 mL dose). In addition to apple polyphenols, there is also evidence that polyphenols found in oranges and pomegranates can also lower postprandial glycaemia, particularly when consumed as juice [64, 65]. The dose of polyphenols used in supplementation studies are within the range reported in typically consumed in fruit (Table 1), providing evidence that the polyphenol content of fruit may contribute to some of the cardiometabolic effects of fruit and fruit juice consumption.

#### Sodium-potassium balance

Dietary sodium and potassium intake are thought to play a key role in the regulation of blood pressure [66]. Whereas dietary sodium is positively associated with blood pressure, dietary potassium is negatively associated with blood pressure. This relationship may be explained by the osmotic potential of sodium and the effects of potassium of sodium sensitivity and excretion [67]. Furthermore, meta-analysis of randomised controlled trials demonstrates that dietary supplementation with potassium for at least 4 weeks can lower blood pressure by a clinically meaningful degree [68]. Whether the dose of potassium delivered by fruit juice and whole fruit can either contribute to, or completely explain, the reduction in blood pressure is unclear. For example, the largest reductions in systolic and diastolic blood pressure with potassium supplementation were − 3.3 (95%CI: -4.9 to -1.6) mmHg and − 2.3 (95%CI: -3.8 to -0.7) mmHg, respectively [68]. These reductions relate to potassium supplementation of ∼ 30 mmol/d (∼ 1170 mg/d) [68], which exceeds the doses of potassium that would be provided by most typical servings of fruit juice (Table 1). These potassium intakes are achievable, however, with intake of whole fruit. Furthermore, meta-regression indicated that the reduction in blood pressure with potassium supplementation is likely to occur at doses smaller than 30 mmol/d [68]. Therefore, potassium content may explain (at least in part) the blood pressure lowering effects of adding fruit juice or whole fruit to the diet.

#### Gut microbiome

The gut microbiome is comprised of bacteria, archaea viruses and eukaryotic microbes residing in the gastrointestinal tract and can play a role in metabolism and immunity. Microbes can liberate short chain fatty acids (SCFAs) from partially and non-digestible polysaccharides, such as acetate (C2), propionate (C3) and butyrate (C4). SCFAs can then act as substrates and signalling molecules regulating aspects of metabolism and inflammation [69]. Evidence generally suggests SCFAs such as propionate can result in favourable cardiometabolic effects such as increased insulin sensitivity in humans [70]. However, rodent data indicate that dietary sugars may increase hepatic *de novo* lipogenesis *via* the production of acetate by the gut microbiome [71]. Whether this is the case in humans with typical sugar intakes is unknown. Whilst ingestion of 20 or 50 g of fructose can induce a detectable increase in serum acetate concentrations in humans [72], ingestion of fructose alone is rare, and co-ingestion of glucose with fructose is thought to potently increase intestinal fructose absorption [73], thereby lowering the amount of fructose made available to the colonic microbiota for fermentation. Accordingly, it is unknown to what extent ingestion of sugars in a simple form (e.g., as SSBs) can provide a substrate for the colonic microbiota in humans.

Sugar sources with a more complex food matrix than SSBs, such as fruit juice and whole fruit could, in theory, influence the gut microbiome *via* changing sugar absorption kinetics such that more fructose enters the colon, or by direct action of other components such as polyphenols and fibre on the gut microbiome. Pectin has been demonstrated to exert a prebiotic effect, with increases in SCFAs following incubation with human faeces [74]. Polyphenols can also be metabolised by the gut microbiota and certain polyphenol metabolites may exert various biological actions. Indeed, it has been suggested that polyphenol metabolites may be responsible for the majority of the biological effects of polyphenols rather than the polyphenols per se [75].

Whether the fibre and polyphenol content of fruit juice and whole fruit (or other aspects of the food matrix) can exert a meaningful change to the human gut microbiome is unclear. One study did demonstrate some changes to the gut microbiome from apples in an in vitro model [14], and non-randomised studies show associations between orange juice consumption and putatively favourable changes in faecal microbiome composition [76, 77]. However, other data from randomised controlled trials, suggest ingestion of two apples per day for 8 weeks is insufficient to detectably alter the human gut microbiome based on faecal samples [78]. When apples have been directly compared to juice, no evidence of differences in the gut microbiome profiles were observed, again based on faecal samples. This includes comparisons of whole apples, apple pomace, clear or cloudy apply juice to control, despite substantial differences in fibre and polyphenol content [4]. This inference was consistent whether based on universal primers targeting 16 S rRNA genes in all bacteria, specific primers for *Bifidobacterium*, faecal pH, or faecal bile acid concentration [4]. One key challenge in the field of gut microbiome research is that faecal sampling is unlikely to accurately reflect the microbiome profile in the gut [79]. Therefore, it can be questioned to what extent the analysis of microbiota from faecal samples reflects changes in the gut microbiota. It is currently unclear to what extent the food matrix can influence the human gut microbiome in vivo and implications for other aspects of physiology and this requires more randomised studies with comprehensive assessments of the gut microbiome.

#### Gut hormones

The gastrointestinal tract secretes a variety of hormones which can contribute to the regulation of metabolism and appetite. These include the incretin hormones glucagon-like-peptide 1 (GLP-1) and glucose-dependent, insulinotropic polypeptide (GIP), which can potential glucose-stimulated insulin secretion and (at least the former) can suppress appetite. Other key hormones secreted by the gastrointestinal tract include cholecystokinin (CCK), peptide tyrosine tyrosine (PYY), and ghrelin. Of these, ghrelin is the only hormone which stimulates appetite.

It has been demonstrated that either comparing apple juice to a sugar-matched control, or by adding various amounts to apple polyphenols to a glucose drink does not increase acute postprandial GIP concentrations, and if anything, can lower GIP concentrations [63, 80, 81]. Furthermore, liquid meals have been demonstrated to increase GLP-1 secretion to a greater extent that solid meals [82]. It is, therefore, unlikely that polyphenols content or solid food form, can improve glycaemic control or reduce body mass via a mechanism of increasing incretin hormone concentrations. The effects of polyphenols and of the physical form of sugars may therefore act on glycaemic control and body mass via other mechanisms. Fibre, on the other hand, may exert some effects *via* gut hormones. SCFAs produced by fermentation of dietary fibre can act on G-protein-coupled receptors (free fatty acid receptor 2 and 3; FFAR2 and FFAR3), which are expressed in the gut epithelium [83]. Non-human animal studies demonstrate that stimulation of these receptors can enhance the release of GLP-1 and PYY from L-cells of the gut [83]. Furthermore, there may be more direct roles of SCFA acting centrally to suppress appetite [83]. The physical form of food could also contribute to food matrix effects on gut hormones. Solid meals can suppress circulating ghrelin concentrations and enhance CCK concentrations to a greater extent than liquid meals [84, 85].

#### Sugar content and type

The type and amount of sugar within foods and drinks can play key roles in the physiological responses to differing food matrices. Except for honey, there is relatively little difference in the sugar content and type of the various food sources compared within this review. Most contain ∼ 10 g sugar per 100 g food (or per 100 mL fluid) and of roughly an equal split between glucose and fructose, either as individual monosaccharides or as the disaccharide sucrose. It is notable that (at least in the EU and UK) portion sizes for these foods differ. Typical portion sizes are 330 mL for SSBs, 150 mL for fruit juice, and one piece of whole fruit (around 80–140 g). Therefore, when provided in these quantities, SSBs would provide ∼ 36 g sugar (of which, ∼ 18 g is fructose), fruit juice would provide ∼ 15 g sugar (∼ 7.5 g fructose) and an apple would provide ∼ 9 g sugar (∼ 4.5 g fructose). Doses of fructose within these ranges may provide an explanation for the effects of sugar sources on glycaemic control if consumed in accordance with serving size guidance.

**Fig. 5:**
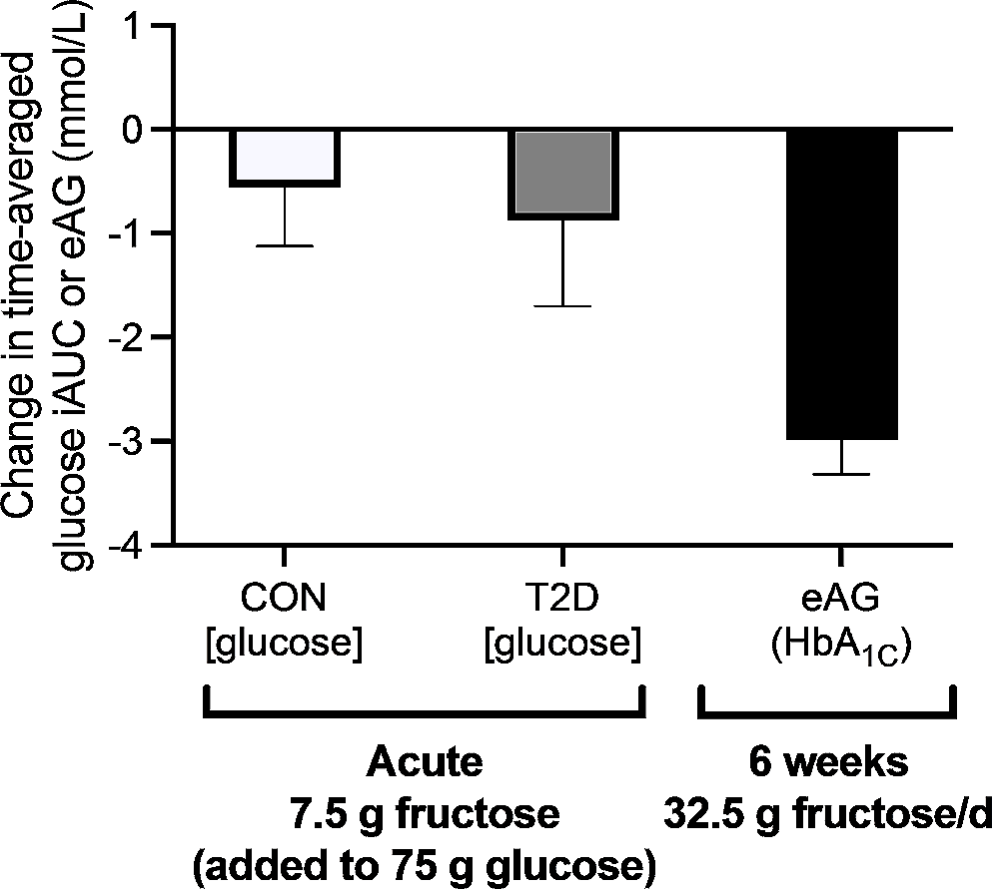
Changes in glucose incremental area under the curve (iAUC) and estimated average glucose concentration (eAG) with acute and longer-term addition of catalytic doses of fructose to the diet (fructose addition vs. non-fructose containing comparator). Data are mean differences and 95%CI from Moore et al., [86] Moore et al. [87] and Sievenpiper et al. [93] HbA1c (%) was converted to estimated average glucose (mmol/L) as per Nathan et al. [94]

Adding a small (7.5 g) dose of fructose to a 75 g oral glucose tolerance test has been shown to lower the glycaemic response in people with and without type 2 diabetes [86, 87]. This has been termed a catalytic dose, as the mechanism by which these doses of fructose can improve glucose tolerance is likely to involve the stimulation of glucokinase translocation. Glucokinase phosphorylates glucose upon entry to the liver and this is a rate-determining step for hepatic glucose metabolism. In the fasted state, most of the hepatic glucokinase is located in the nucleus, bound to the glucokinase regulatory protein (GKRP). GKRP preferentially interacts with glucokinase when GKRP is bound to fructose-6-phosphate. However, fructose-1-phophate competitively inhibits the binding of GKRP to fructose-6-phosphate, thereby releasing glucokinase from GKRP to allow translocation [88]. Increasing fructose availability in the portal vein of dogs has been shown to increase hepatic fructose-1-phosphate concentrations by more than 170% [89]. It should be noted that two more recent studies have failed to replicate the acute effects of catalytic doses of fructose on glucose tolerance [90, 91]. The reasons for this heterogeneity across studies is currently unclear, although suggestions of endogenous fructose production from the dose of glucose provided, and blood sampling methods (venous vs. arterialised [92]), have been suggested to play a role [91]. Longer-term studies do, however, generally support the concept of catalytic doses of fructose lowering glycaemic responses, with meta-analyses demonstrating reductions in HbA1c and fasting glucose concentrations when a median intake of fructose of 32.5 g/d is ingested over a median of 6 weeks [93] (Fig. 5).

### Fat, protein and water content

The fat and water content of foods are primary factors dictating energy density, whereas all three of these components could also have more direct effects on physiology [95]. Whilst fruit contains little fat and protein, other sources of sugars often contain these additional macronutrients in quantities that are biologically relevant. Both protein and fat can contribute to slowing gastric emptying rates and increasing gut hormone secretion [96, 97], although this depends on the specific type of protein added to a meal [98]. However, in contrast to the physical structure, whereby gastric emptying rates can be slowed without altering the energy content of a food, the addition of fat or protein to a food will increase the energy content and therefore the slowing of gastric emptying does not necessarily result in a reduction in net energy intake [96]. The slowing of gastric emptying and gut hormone secretion can, however, contribute to lowering of glucose concentrations in response to a meal [96, 97, 99]. Whey protein can also acutely lower blood pressure by ∼ 3 mmHg [100], which could be due to the insulinaemic properties or more direct actions of specific peptides. Accordingly, the content and type of fat and protein within sugar-containing foods could alter the physiological responses in a variety of ways, including altering gastric emptying, gut hormone and insulin responses, which in turn may lower glucose concentrations and blood pressure. However, these responses will depend on the specific interactions between the type and amount of protein and fat with the other properties of a food and therefore the wide range of sugar containing foods which also contain protein and fat are likely to produce heterogenous physiological responses.

### Other considerations

The present review has focussed on the effects of sugar sources on cardiometabolic health outcomes, yet consideration should also be made to the role of energy balance and energy turnover. Diets high in sugars can lead to an increase in energy intake, at least in part, due to the energy density of the diet [33]. Positive and negative energy balance play a role in mediating cardiometabolic health in tandem with changes in diet composition [101]. Furthermore, even within a similar degree of energy surplus, energy turnover plays a role in modulating the effects of overfeeding [102]. This also has specific relevance to sugar metabolism since daily exercise can prevent the increase in triglycerideaemia seen with fructose overfeeding, even when the energy surplus is matched [103]. These modulatory effects of energy balance and energy turnover on the effects of dietary sugars may involve hepatic glycogen metabolism [104]. Accordingly, energy balance and physical activity status should be considered when interpreting the effects of sugar sources on cardiometabolic health.

## Conclusion

**Fig. 6:**
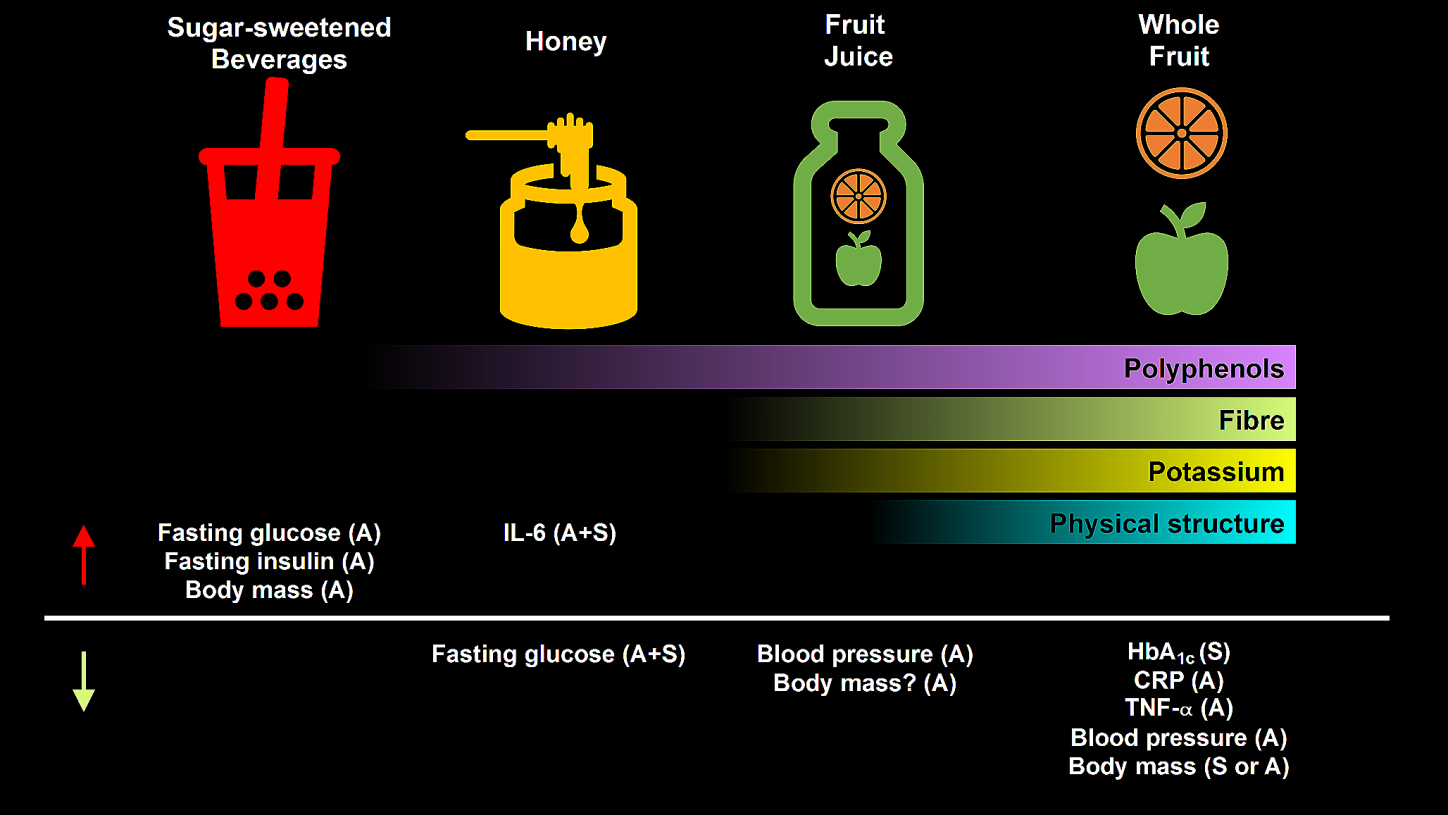
An overview of the differences between important sugar sources in nutrient composition and physiological responses when these are either added as excess energy to the diet (A), substituted for other energy sources (S) or added and substituted (A + S) to the diet

Sugar sources vary widely in their food matrices, which includes differences in the nutritional composition and physical structure. SSBs could be considered as having a “simple” physical structure, being in liquid form and with few additional nutrients (i.e., negligible content of polyphenols, fibre, and potassium). Honey may contain a small quantity of polyphenols, but it is unclear if these are in sufficient quantities to exert meaningful physiological effects. Fruit juices typically contain relatively higher concentrations of polyphenols, fibre, and potassium, especially when expressed per g of sugar, and whole fruit has the additional complexity of a solid (or semi-solid) physical structure and a higher fibre content. These characteristics may contribute to the physiological effects of consuming these sugar sources, whereby meta-analyses demonstrate the addition of SSBs to the diet can increase fasting glucose and insulin concentrations in addition to body mass. In contrast, honey appears to decrease fasting glucose concentrations but increase some markers of inflammation, the relevance of which is currently unclear. Addition of fruit juices to the diet may increase fasting glucose and HbA_1c_, but can lower blood pressure and body mass and, when substituted into the diet, can lower some markers of systemic inflammation. Finally, the addition of whole fruit to the diet can lower markers of systemic inflammation, blood pressure and body mass, and with substitution, can improve markers of glycaemic control (Fig. 6). Therefore, from a cardiometabolic health standpoint, whole fruit can consistently and reliably improve markers of cardiometabolic health and are a cornerstone of a healthy dietary pattern.

There is currently relatively little direct comparison of sugar sources on cardiometabolic markers or health outcomes. Of the currently available data on direct comparisons of whole fruit *versus* fruit juice, there is no clear evidence for meaningful differences in glycaemic control, inflammation, or blood pressure. There is, however, consistent evidence that whole apples can lower plasma low-density lipoprotein cholesterol concentrations compared with fruit juice. Acute appetite responses suggest whole fruit increases satiety to a greater extent than fruit juice, but comparative changes in body mass and composition have not been studied in detail. Medium-term interventions with daily fruit juice have not led to consistent, significant overall body weight or compositional changes. Further research on direct comparisons of sugar sources and on complex foods with multiple ingredients and difference structures, would contribute to a better understanding of the causal role of food matrix on the cardiometabolic responses to sugar consumption.
